# Iterative optimization algorithm with structural prior for artifacts removal of photoacoustic imaging

**DOI:** 10.1016/j.pacs.2025.100726

**Published:** 2025-04-26

**Authors:** Yu Zhang, Shuang Li, Yibing Wang, Yu Sun, Tingting Huang, Wenyi Xiang, Changhui Li

**Affiliations:** a Department of Biomedical Engineering, College of Future Technology, Peking University, Beijing, China; b School of Electrical and Electronic Engineering, Nanyang Technological University, , Singapore, Singapore; c National Biomedical Imaging Center, Peking University, Beijing, China

**Keywords:** Iterative regularization method, Structural prior, Probability matrix, Random partial array, Sparse sensor distribution, Artifact removal, Imperfect distributed arrays

## Abstract

In reality, photoacoustic imaging (PAI) is generally influenced by artifacts caused by sparse array or limited view. In this work, to balance the computing cost and artifact removal performance, we propose an iterative optimization method that does not need to repeat solving forward model for every iteration circle, called the regularized iteration method with structural prior (RISP). The structural prior is a probability matrix derived from multiple reconstructed images via randomly selecting partial array elements. High-probability values indicate high coherency among multiple reconstruction results at those positions, suggesting a high likelihood of representing true imaging results. In contrast, low-probability values indicate higher randomness, leaning more towards artifacts or noise. As a structural prior, this probability matrix, together with the original PAI result using all array elements, guides the regularized iteration of the PAI results. The simulation and real animal and human PAI study results demonstrated our method can substantially reduce image artifacts, as well as noise.

## 1 Introduction

PAI has emerged as a promising imaging modality that combines the high structural resolution and imaging depth of ultrasound with the rich optical contrast of biological tissues [1], [2], [3]. This hybrid imaging technique has found broad applications in biomedical imaging, particularly in scenarios such as vascular imaging [4], tumor detection [5], [6], and brain imaging [7]. However, in reality, the PAI system often does not have enough sensors or acquisition angles to fulfill the exact reconstruction condition, causing artifacts to degrade the quality of reconstructed results. This challenge becomes even more serious for those three-dimensional (3D) PAI systems.

M. Pramanik [8] reconstructs multiple low-resolution images from randomized detection points data and computes an artifact score matrix to identify and suppress artifacts. However, the quantification of this method is problematic due to the fact that the artifact score matrix is obtained from a variant of the coefficient of variation. A way to increase image quality is to use the weighting factors. Various weighting algorithms have been proposed so far, such as coherence factor (CF), spatiotemporal coherence factor (STCF) [9], Hilbert-based CF (H-CF) [10]. These methods provide images with high resolution and high contrast but some low-intensity photoacoustic absorbers may also be suppressed [11] and their performance in circular-view or hemispherical-view configurations has not been evaluated yet. Some post-processing methods, such as Block-Matching and 4D/3D Filtering (BM4D/BM3D) [12], [13], Noise2Noise [14], have achieved certain progress in noise reduction, but they are almost incapable of removing artifacts in photoacoustic images. Meanwhile, researchers have developed iterative reconstruction (IR) methods to enhance the quality of the results. Wang et al. [15], [16] demonstrated that iterative penalized least-squares methods, constructed on discrete-to-discrete imaging models with expansion functions over different fixed structural grids, and using quadratic smoothing or total variation (TV) norm constraints, could significantly improve the performance of 3D PAI systems for small animals. Similarly, Huang et al. [17] proposed forward and backward operators based on k-space pseudospectral methodologies. While these IR techniques have shown excellent results, the computing burden in both time and memory consumption is still a serious challenge for their implementations, especially for large-scale 3D PAI [18]. Researchers have, therefore, explored novel alternatives. Arridge et al. addressed this by introducing efficient numerical implementations of adjoint operators for PAI reconstruction and applying compressed sensing via Bregman iteration to reduce sensor requirements [19]. However, their method was computationally expensive and restricted to voxel counts on the scale of $10^{6}$. Shang et al. [20] took a different approach by building a forward model using directly measured graphite point sources, but the inefficiencies in optimization and inaccuracies in the model itself limited its practicality. Deep learning-based methods for PAI reconstruction improve computational efficiency and image quality [21], [22], [23] but depend on large, specialized datasets that are hard to obtain and struggle to generalize across different imaging systems or conditions, limiting their clinical applicability.

Unlike the traditional IR algorithms that demands repeating solving forward model or other complex process for every iteration circle, here, we propose an optimization method for PAI image reconstruction that does not need solving the forward model. The key idea of this method is based on the fact that artifact are more sensitive to detection configurations, i.e., pixel values of artifacts would fluctuate a lot when alternating detector number or detection geometry, while true PA signals are relatively stable. Therefore, we construct a probability matrix based on a large number of reconstructed images from subsets of randomly picked array elements. As a structural prior, this probability matrix guides the iteration and regularization of the originally reconstructed result using full array elements. This method can be applied to any reconstruction algorithms, such as DAS or UBP, as well as any detection configurations. By leveraging the structural prior based on possibility matrix and iteratively refining reconstruction results, this method can substantially improve image quality while keeping high quantitative accuracy. Both simulation and experimental results demonstrate the effectiveness of the proposed method in reducing artifacts under sparse-view conditions while preserving critical structural information in PAI reconstructions.

## 2 Method

This part is mainly divided into two parts. The first part is the construction of probability matrix generated by subsets of randomly selected detectors, which provides a structural prior knowledge for subsequent regular iteration; The second part is the specific iteration optimization scheme.

### 2.1 Construction of the probability matrix

#### 2.1.1 Random selection strategy

For a PAI array system with the element number N and temporal sampling number M, the data dimension of the recorded signal is N×M. In addition to using all N elements to do image reconstruction, we generate k subsets from original N elements, and each subset consists of s(s<N) randomly selected elements. Then, there are k images can be reconstructed use these subsets, naming $R_{1},R_{2},\ldots ,R_{k}$. (The choices of s and k are described in detail in subsequent paragraphs.)

#### 2.1.2 Probability matrix

The probability matrix is defined as P.

(1) $$P=D⊙\int_{v_{\min}}^{v_{\max}}\Phi \left(v|\mu ,\sigma \right)⊙u\left(D-v\right)dv$$

where, u(x) is the unit step function, and D is calculated by

(2) $$D=\frac{{\left(\frac{1}{k}\left(R_{1}+R_{2}+\cdots +R_{k}\right)\right)}^{2}}{\frac{1}{k}\left(R_{1}^{2}+R_{2}^{2}+\cdots +R_{k}^{2}\right)}=\frac{{\left(R_{1}+R_{2}+\cdots +R_{k}\right)}^{2}}{k\left(R_{1}^{2}+R_{2}^{2}+\cdots +R_{k}^{2}\right)}$$

$v_{min}=\min\left(D\right),\;v_{max}=\max\left(D\right),\;\mu =\left(v_{max}+v_{min}\right)/2,\;\sigma =\left(v_{max}-v_{min}\right)/6$, and Φ(v|μ,σ) is the cumulative distribution function of the normal distribution.

(3) $$\Phi \left(v|\mu ,\sigma \right)=\frac{1}{\sigma \sqrt{2\pi }}\int_{-\infty }^{v}e^{\frac{-{\left(t-\mu \right)}^{2}}{2\sigma^{2}}}dt$$

In order to have a more intuitive physical meaning for the subsequent regular iteration process, we use normalized $P_{norm}$ $\left(0<P_{norm}<1\right)$.

(4) $$P_{norm}=\frac{P-\min\left(P\right)}{\max\left(P\right)-\min\left(P\right)}$$

The dimensions of P,D,Φ and R are the same as the reconstructed image, either 2D or 3D. The Matrix $P_{norm}$ is a probabilistic representation of the true data. The function integrates the local property D, which reflects the concentration of the data $R_{1},R_{2},\ldots ,R_{k}$. This makes D a measure of the data stability, where higher values indicate more stability. The introduction of the Gaussian cumulative distribution-based weighting factor Φ(v|μ,σ) combines local signal intensities with global distribution characteristics. Strong signal regions receive higher weights, while weaker signals are appropriately suppressed but not entirely discarded. It ensures that the probability matrix is distributed as closely as possible towards the two extremes of 0 and 1, thereby providing beneficial support for the convergence of subsequent iterative processes and the final results. The Gaussian distribution, determined by its mean μ and standard deviation σ, dynamically reflects the global distribution pattern of signals. Consequently, this weighting approach enhances signal saliency and selectively preserves significant regions, achieving more robust and efficient reconstruction. This integral effectively blends the local signal characteristics with global weighting, enabling the assignment of weights that emphasize real signals while preserving the overall smooth and consistent distribution across the signal space.

### 2.2 Regularized iteration optimization

In this section, we will use the probability matrix $P_{norm}$ obtained in the previous section as a structural prior and design a suitable loss function for regular iterative optimization of the original reconstruction results of the entire array signals.

#### 2.2.1 Structural prior and loss function

##### Structural prior.

The feasibility of using $P_{norm}$ as a structural prior lies in the fact that it directly provides the probability distribution information of each voxel or pixel region, which can be used to indicate the model’s tendency to strengthen certain regions (high probability regions) in the reconstruction results and its inhibition demand for other regions (low probability regions). This prior information can effectively guide the optimization process, making the result more in line with the expected structural characteristics, while reducing the impact of noise or artifacts. The rational constraint of shape information is realized mathematically, and the optimization stability of shape consistency is improved.

##### Loss function.

The original reconstructed with all array signals is $R_{N}$ which is to be optimized. The result of each iteration optimization is $R_{op}$. To enhance the generalizability of the algorithm and reduce the need for extensive parameter adjustments during application, $R_{N}$ and $R_{op}$ are normalized to the range of [0,1]. The loss function consists of two terms: data consistency loss term [24] and regularization loss term [25].

The data consistency loss term is represented by $Loss_{dc}$.

(5) $$Loss_{dc}={\left\|R_{N}-R_{op}\right\|}_{F}^{2}$$

Its corresponding gradient is $gradient_{dc}$.

(6) $$gradient_{dc}=\frac{\partial {Loss}_{dc}}{\partial R_{op}}=2\times \left(R_{N}-R_{op}\right)$$

The role of the data consistency loss term is to ensure that the optimized results do not deviate too much from the original reconstruction result $R_{N}$, which is still approximate to the true image. Thereby maintaining physical consistency and quantitative accuracy.

The regularization loss term is represented by $Loss_{rg}$.

(7) $$Loss_{rg}={\left\|\left(1-P_{norm}\right)⊙R_{op}\right\|}_{F}^{2}$$

Its corresponding gradient is $gradient_{rg}$.

(8) $$gradient_{rg}=\frac{\partial {Loss}_{rg}}{\partial R_{op}}=2\times \left(1-P_{norm}\right)⊙R_{op}$$

The significance of regularization loss term is to use the structural prior knowledge to constrain the optimization process. As we mentioned in the previous section, in the actual reconstruction, the results obtained by using the traditional reconstruction algorithm are accompanied by noise and artifacts, which degrade reconstruction results. By combining the prior information provided by the prior probability distribution $P_{norm}$, the regularization term can actively suppress the low probability region, thereby reducing both of artifacts and noise. The regularization term guides the optimal solution to cluster in the high probability region (these regions are more in line with the prior physical knowledge).

Due to the regularization introduced in the optimization process, the $R_{op}$ of the optimization result may deviate from the original data, and even lose the important features of original signals. Therefore, the data consistency loss term imposes constraints in the optimization process, forcing the optimization results to be consistent with the original reconstructed data as much as possible, so that the optimized volume data can gradually de-noise and enhance the structure while still reflecting the main features and information of the input data. This is a balancing mechanism that avoids distortion caused by excessive regularization and strengthens the physical confidence and data integrity of the optimized results, which is essential for quantitative accuracy.

#### 2.2.2 Optimizer selection and regularized iteration process

##### Optimizer selection.

We selected the Adam optimizer (Adaptive Moment Estimation) as our optimization algorithm. Adam is a first-order gradient-based optimization algorithm that combines the advantages of momentum and root mean square propagation (RMSProp) [26], making the optimization process more stable and efficient in complex reconstruction tasks. Specifically, the momentum mechanism reduces oscillations and gradient noise by applying an exponentially weighted average to the gradients, while RMSProp’s adaptive learning rate ensures proper adjustments of the learning rates across different variable dimensions. Additionally, Adam incorporates bias correction, which rectifies the biases during early iterations, thereby improving stability. Overall, with its fast convergence and adaptive features, the Adam optimizer meets the requirements of our task.

##### Regularized iteration process.

The following is the specific algorithm pipeline of iterative optimization:

**Figure:**
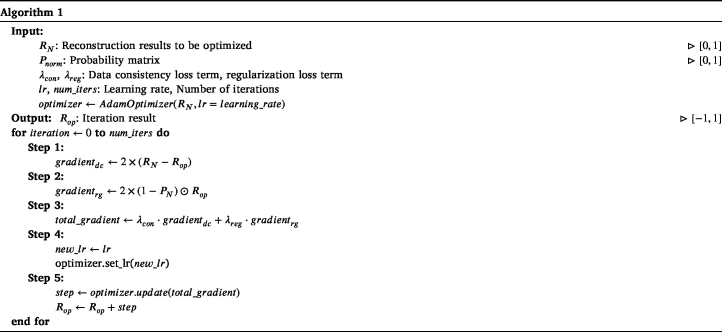

## 3 Results

In this session, we use simulation data and in vivo animal experiment data to validate the performance of RISP algorithm.

### 3.1 Simulation studies

#### 3.1.1 3D PAI image reconstruction with a hemispherical array system

**Fig. 1:**
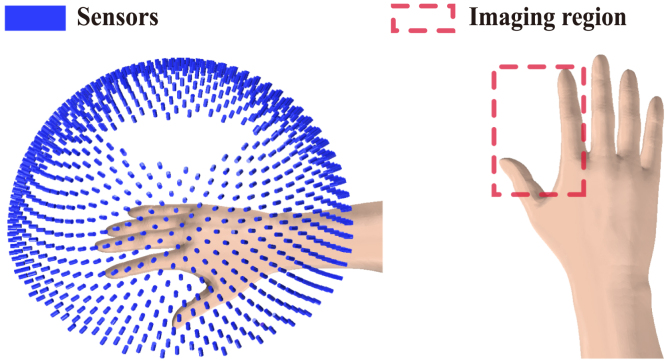
The position of the hand and PAI array sensors.

We first used simulated human hand vessels as the target tissue to demonstrate the high performance of the RISP algorithm. The simulated data is based on the 3D PA imaging results of human peripheral hand vessels from another work [27], which was used as the ground truth. At a grid point spacing of 0.2 mm, the total grid size of the imaging area is 128 × 320 × 256 (25.6 mm × 64.0 mm × 51.2 mm). The PAI system is a semi-spherical array system with the radius of 4 cm, and a hole with a height of 1 cm is opened at the top for the laser illumination. There are a total of 1024 evenly distributed array elements (Fig. 1 is the imaging setup). Then we obtained PA signals collected by these 1024 arrays through forward propagation of the K-Wave toolbox [28].

##### Comparison of results.

**Fig. 2:**
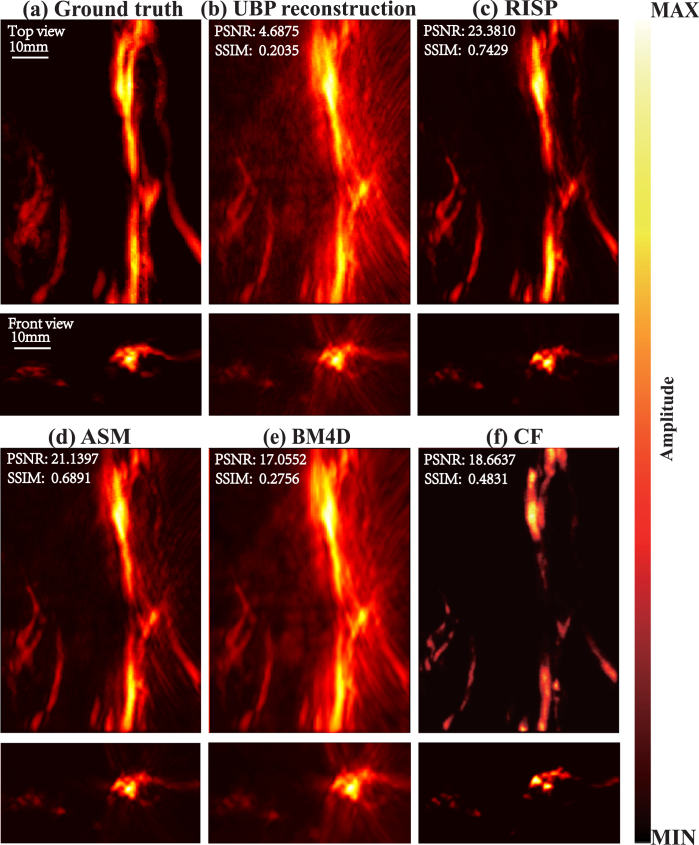
Comparison of 3D photoacoustic reconstruction results with 1024 hemispherical array system. (a) Finger’s top-view MAP, and front-view MAP. (b) UBP results. (c) RISP results. (d) ASM results. (e) BM4D results. (f) CF results.

**Fig. 3:**
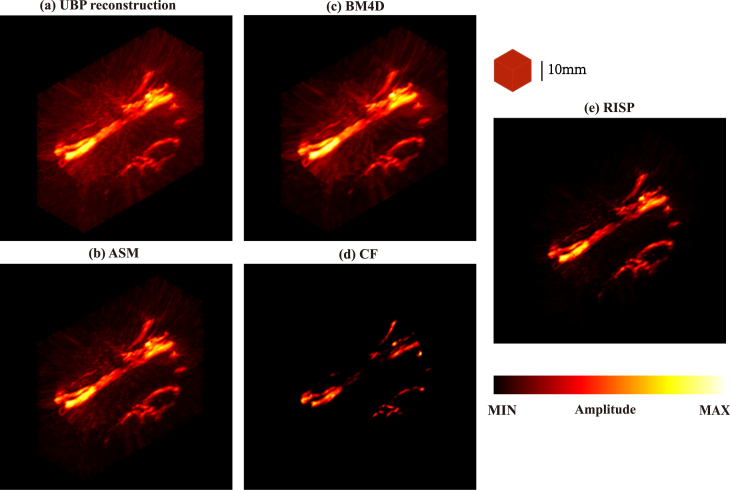
Comparison of 3D photoacoustic reconstruction results with 1024 hemispherical array system in a 3D view. (a) UBP results. (b) ASM results. (c) BM4D results. (d) CF results. (e) RISP results.

To better present the results, all final results are presented after taking their absolute values. The method we proposed is compared with the method based on artifact score matrix (ASM) [8], Block-Matching and 4D/3D Filtering (BM4D/BM3D), and the method based on coherence factor (CF). The ground truth is presented in Fig. 2(a), while the reconstruction results are in Fig. 2(b–f). For each set of images, the upper and lower subimages correspond to the top-view maximum amplitude projection (MAP) and the front-view MAP of the 3D reconstruction, respectively. In Fig. 2(b), the reconstruction results obtained via the UBP algorithm display prominent artifacts. As shown in Fig. 2(c), the RISP algorithm significantly reduces the artifacts in the UBP results. Furthermore, in the 3D view (Fig. 3), the improvements after applying the RISP algorithm are clearly observable. The results of ASM and BM4D exhibit more artifacts. Although the CF results are almost free of artifacts, lots of actual vascular information is also removed. We also performed the peak signal-to-noise ratio (PSNR dB) and the structural similarity index measure (SSIM) of the reconstruction results before and after using RISP to optimize and using other methods and all results are normalized. The results are listed in Fig. 2. It further demonstrates the effectiveness of the RISP optimization.

**Fig. 4:**
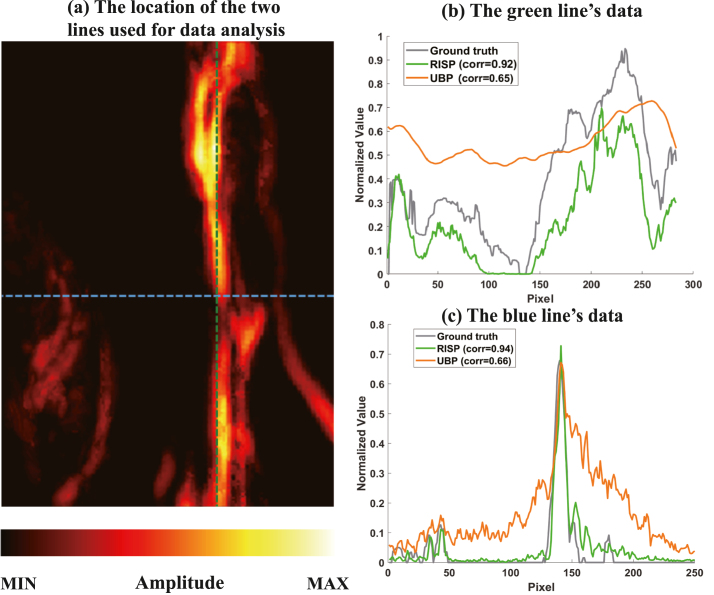
(a) Finger image with two marked lines. (b) Comparison along the green line. (c) Comparison along the blue line.

Specifically, we extracted data along two lines. The voxel intensity values along the lines are presented in Fig. 4. In Fig. 4(b), it is evident that, in regions containing finger information, the intensity curve of the RISP-optimized result aligns closely with the ground truth, demonstrating superior consistency compared to the UBP-only reconstruction. The correlations before and after using RISP are 0.65 and 0.92. In Fig. 4(c), it can be seen that in regions lacking finger data, the noise and artifact levels in the RISP-optimized results are significantly closer to zero, while the noise and artifact levels in the UBP-only reconstruction are considerably higher. The correlations before and after using RISP are 0.66 and 0.94.

In conclusion, in the simulation experiments, the reconstruction results obtained using the UBP algorithm and further optimized by the RISP algorithm demonstrated excellent performance, particularly in artifact suppression, noise reduction, and the preservation and representation of true structural information.

##### Parameters selection and computing environment.

In this simulation experiment, the computing environment and specific values of parameters we used are in Table 1.

We used single GeForce RTX 4090 and the CPU type is AMD® Epyc 9354 32-core processor × 128. In UBP algorithm reconstruction, we use Taichi [29], [30] for GPU acceleration. For the reconstruction with PA signal size of 50 × 4096 and region size of 128 × 320 × 256, the time consumption is about 0.5 s. The computation of the probability matrix takes about 35 s, and the entire iteration process takes about 10 s. So the entire RISP optimization process took less than a minute and 10 s (0.5s×50+35s+10s). The choice of s is generally about one-tenth of the total number of elements.

**Table 1:** 3D PAI image reconstruction with a hemispherical array system.

Parameters	Value
N	1024
M	4096
s	50
k	50
$\lambda_{con}$	0.10
$\lambda_{reg}$	0.90
lr	0.001
num_iters	50

#### 3.1.2 2D PAI image reconstruction with PAI ring system

**Fig. 5:**
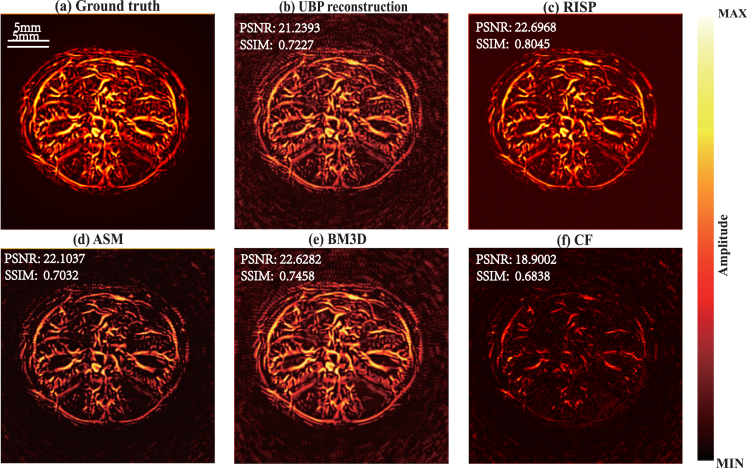
Comparison of 2D photoacoustic reconstruction results with PAI ring system. (a) Ground truth. (b) UBP results. (c) RISP results. (d) ASM results. (e) BM3D results. (f) CF results.

In this simulation study, the PAI system is a circular ring array system having a radius of 5 cm and 256 elements. The simulated PA temporal signal is calculated by the K-Wave toolbox. The simulated phantom is a PA vascular structure from another literature [31] (Fig. 5(a)). At a grid point spacing of 0.05 mm, the total grid size of the imaging area is 512 × 512 (25.6 mm × 25.6 mm).

##### Comparison of results.

For better display effect, all final results are presented after taking their absolute values. From Fig. 5, the artifacts significantly decrease after optimization by the RISP algorithm. Details of blood vessels are displayed more clearly. The results of ASM and BM3D exhibit more artifacts. Although the CF results are almost free of artifacts, a lot of information we want is also removed. The PSNR(dB) and SSIM results are shown in Fig. 5. Both the artifacts around the internal small blood vessels and the overall peripheral artifacts after using RISP are effectively suppressed.

##### Parameters selection and computing environment.

The specific values of the parameters we used are listed in Table 2.

In this simulation experiment, we only used CPU. And the CPU type is 13th Gen Intel(R)Core(TM)i5-13500H. In UBP algorithm reconstruction, for the reconstruction with PA signal size of 50 × 2048 and region size of 512 × 512, the time consuming is about 0.50s. The computation of the probability matrix takes about 30 s, and the entire iteration process takes about 10 s. So, the entire RISP optimization process took about 3 min (0.50s×100+30s+10s).

**Table 2:** 2D PAI image reconstruction with PAI ring system.

Parameters	Value
N	256
M	2048
s	80
k	100
$\lambda_{con}$	0.20
$\lambda_{reg}$	0.80
lr	0.001
num_iters	150

### 3.2 Validation by human PAI studies

#### 3.2.1 3D PAI image reconstruction with synthetic matrix array system

Then in human experiment, the data of the arm was acquired by Li’s work [27] using synthetic matrix array system. The system used a non-focusing linear array (customized by Imasonics, France) to receive PA signals. The linear array has 256 elements with a pitch of 0.5 mm and a kerf of 0.1 mm, i.e., a total length of 12.8 cm. The center frequency of the ultrasonic array is 3.5 MHz with over 80% bandwidth. PA signal is amplified 1500 times via self-built two-stage amplifier, and then received by a data acquisition system (Marsonics DAQ, Tianjin Langyuan Technology Co., Ltd. China) at 40 MHz sampling rate.

In the original work, a linear array consisting of 256 elements was moved 2969 steps, with a step size of 0.1 mm for each movement, resulting in a large-scale synthetic matrix of 256 × 2969 elements. To create a sparse-view setup, we took 26 rows equally spaced within the range of 5–15 cm in the direction along the arm.

#### 3.2.2 Comparison of results

**Fig. 6:**
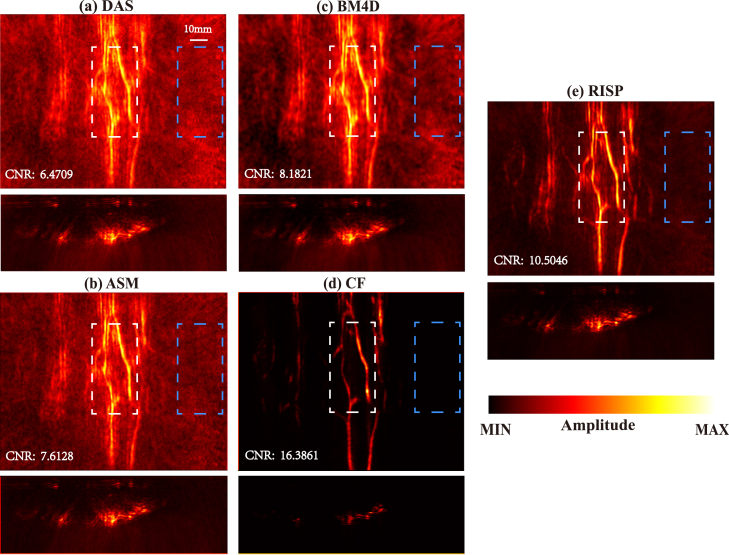
Comparison of 3D photoacoustic reconstruction results with synthetic matrix array system. (a) DAS results. (b) ASM results. (c) BM4D results. (d) CF results. (e) RISP results.

**Fig. 7:**
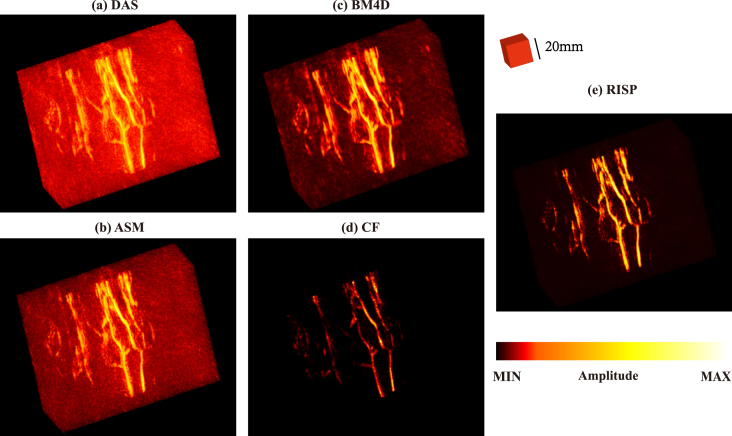
Comparison of 3D photoacoustic reconstruction results with synthetic matrix array system in a 3D view. (a) DAS results. (b) ASM results. (c) BM4D results. (d) CF results. (e) RISP results.

The detailed comparison of results before and after optimization is presented in Fig. 6, Fig. 7. To better show the performance of each method, we used a consistent adjusted colorbar for all results in the 3D view.

We compared the hand imaging results using RISP, ASM, BM4D and CF. It can be seen that the RISP removes more artifacts and noise. In addition, we used the area enclosed by the white rectangle as the target signal region and the area enclosed by the blue rectangle as the noise region to calculate the contrast-to-noise ratio (CNR dB). The results are listed in Fig. 6. Although the results of CF have almost no artifacts and noise and CNR is the highest, a lot of vascular information is also removed.

#### 3.2.3 Parameters selection and computing environment

In this animal experiment, the specific values of the parameters we used are in Table 3.

We used single GeForce RTX 4090 and the CPU type is AMD® Epyc 9354 32-core processor × 128. In UBP algorithm reconstruction, we use Taichi for GPU acceleration. For the reconstruction with PA signal size of 30 720 × 2048 and region size of 625 × 600 × 250, the time consuming is about 10.2 s. For the reconstruction with PA signal size of 4000 × 2048 and region size of 625 × 600 × 250, the time consuming is about 3.5 s. The computation of the probability matrix takes about 140 s, and the entire iteration process takes about 90 s. So the entire RISP optimization process took less than 7 min (3.5s × 50+140s+90s).

**Table 3:** 3D PAI image reconstruction with synthetic matrix array system.

Parameters	Value
N	30 720
M	2048
s	4000
k	50
$\lambda_{con}$	0.10
$\lambda_{reg}$	0.90
lr	0.001
num_iters	50

## 4 Discussion

In this study, we focus on demonstrating the optimization effectiveness of the RISP algorithm applied to the reconstruction results of the UBP and DAS multiplying algorithms. Through a series of specific experiments, the RISP algorithm has shown exceptional performance. Moreover, the unique concept of the RISP algorithm lies in optimizing existing imaging results rather than directly reconstructing images within the signal domain. This unique characteristic grants the algorithm broad applicability, enabling effective suppression of noise and artifacts caused by transducer-related factors (e.g., transducer distribution and other system properties). Importantly, the proposed RISP optimization is a general strategy, which does not depend on a specific reconstruction algorithm.

However, the performance of the RISP is influenced by the quality of the structural prior, which optimizes the result reconstructed by all array elements, therefore the RISP method is not suitable for extremely sparse or limited-angle cases in which the artifacts dominate the prior. In future, we will continue to optimize the speed and quality of RISP. Besides, we will also explore it in other potential biomedical imaging fields beyond PAI, such as ultrasound imaging and computed tomography (CT).

## CRediT authorship contribution statement

**Yu Zhang:** Writing – original draft, Validation, Software, Methodology, Investigation, Formal analysis. **Shuang Li:** Resources, Methodology. **Yibing Wang:** Software, Methodology, Formal analysis, Conceptualization. **Yu Sun:** Resources. **Tingting Huang:** Visualization. **Wenyi Xiang:** Software, Methodology. **Changhui Li:** Writing – review & editing, Methodology, Funding acquisition.

## Declaration of competing interest

The authors declare that they have no known competing financial interests or personal relationships that could have appeared to influence the work reported in this paper.

## Data Availability

Data will be made available on request.
